# The Future of Bacteriophage Therapy Will Promote Antimicrobial Susceptibility

**DOI:** 10.1128/mSystems.00218-21

**Published:** 2021-07-20

**Authors:** Olivia Williams Barber, Iria Mañas Miramontes, Manu Jain, Egon A. Ozer, Erica M. Hartmann

**Affiliations:** a Department of Civil and Environmental Engineering, Northwestern University, Evanston, Illinois, USA; b Division of Pulmonary and Critical Care Medicine, Department of Medicine, Northwestern University Feinberg School of Medicine, Chicago, Illinois, USA; c Department of Medicine, Division of Infectious Diseases, Northwestern University Feinberg School of Medicine, Chicago, Illinois, USA; University of Wisconsin-Madison

**Keywords:** *Pseudomonas aeruginosa*, antibiotic resistance, bacteriophage therapy, evolutionary rescue

## Abstract

Rising antimicrobial resistance severely limits efforts to treat infections and is a cause for critical concern. Renewed interest in bacteriophage therapy has advanced understanding of the breadth of species capable of targeting bacterial antimicrobial resistance mechanisms, but many questions concerning ideal application remain unanswered. The following minireview examines bacterial resistance mechanisms, the current state of bacteriophage therapy, and how bacteriophage therapy can augment strategies to combat resistance with a focus on the clinically relevant bacterium Pseudomonas aeruginosa, as well as the role of efflux pumps in antimicrobial resistance. Methods to prevent antimicrobial efflux using efflux pump inhibitors and phage steering, a type of bacteriophage therapy, are also covered. The evolutionary context underlying antimicrobial resistance and the need to include theory in the ongoing development of bacteriophage therapy are also discussed.

## INTRODUCTION

Antimicrobial resistance (AMR) is a major threat to modern medical advancements. Although antimicrobial drugs create a hostile environment for bacteria, antimicrobials cannot change their mechanism of action, while bacteria can evolve defenses and experience evolutionary rescue. Evolutionary rescue causes even new antimicrobials to often become ultimately ineffective against the most resistant bacteria (1). The rise of resistance requires us to develop novel solutions to combat infection. Bacteriophage, or phage, therapy uses viruses that infect bacteria to treat human infections, often in conjunction with antimicrobial treatment (2–4). Phage not only provide an alternative method to kill pathogens and an additional selective pressure, but also coevolve with their bacterial hosts.

While phage therapy was used widely prior to the discovery of antibiotics (5), the initial availability of antibiotics exclusively to the Allies in World War II, confusion about the science of phages, and suspicion of Soviet scientific results after the war led to the lack of adoption outside the former Soviet Union (3). In Tbilisi, Georgia, the George Eliava Institute of Bacteriophage, Microbiology and Virology has been conducting phage therapy research and treatment for almost a century (6). Although Georgia, Russia, and a few other Eastern European countries widely use phage-based treatments (7), there are no Food and Drug Administration (FDA)-approved phage therapies, and use in the United States is reserved for compassionate treatment (8).

One type of phage therapy that considers evolutionary pathways of resistance is phage steering. Phage steering intentionally combats resistant infections by forcing bacteria to resist the selection pressure of either phage or antimicrobials (9). Currently, most phage steering applications use natural phages that are prescreened for efficacy against a specific bacterial target. Using phage steering to target AMR mechanisms can potentially extend the life span of current antimicrobials by anticipating that bacteria will develop resistance (10). AMR in Pseudomonas aeruginosa is particularly concerning because it is a common cause of acute and chronic infections in humans and is predisposed to the development of resistance (11, 12).

The following review focuses on countering resistance in P. aeruginosa and how bacteriophage therapy can improve existing approaches. A brief overview of bacterial resistance methods, including efflux pumps, and the evolutionary theory behind antimicrobial resistance is provided. How these mechanisms influence bacteriophage therapy, and the potential repercussions, will be considered along with improvements to phage treatment. The use of phage steering and how it enhances current strategies targeting the bacterial efflux system, as well as its use with other phage and antimicrobial treatments, will be highlighted.

## BACTERIA USE BOTH BIOCHEMICAL AND PHYSICAL STRATEGIES TO RESIST ANTIMICROBIALS

Resistance to toxic substances is an intrinsic protective strategy that has long predated clinical use of antimicrobials (13). Increased clinical, industrial, and agricultural use of antimicrobials such as β-lactams, aminoglycosides, and quinolones has promoted multidrug resistance in many clinically relevant bacterial organisms, including P. aeruginosa (14).

Bacteria employ many resistance strategies such as production of degradative enzymes, formation of biofilm structures regulation or alteration of outer membrane proteins, and antimicrobial secretion through efflux pumps. Bacteria can inactivate antimicrobials using enzymes like adenylyltransferase ANT(′′)-Ia, which results in resistance to three antimicrobials in the aminoglycoside family (gentamicin, tobramycin, and kanamycin) (15). However, the mechanisms and regulation of enzyme-induced AMR, such as AmpC β-lactamase overproduction (16) or the influence of protein CATB7 in chloramphenicol resistance (17), are not always well understood.

Biofilm growth is often associated with increased AMR compared to that of planktonic growth of bacteria, in part because not all members of the biofilm need to express drug-inactivating enzymes to protect the community as a whole. Commonly, susceptible bacteria will reside alongside drug-resistant bacteria within the structure, creating an internal diversification of bacterial activities, which can be an impediment to successful antimicrobial therapy (18). While biofilms are considered generally recalcitrant to antimicrobials (19, 20), not all studies have found significant differences in resistance (21).

Both biofilm growth and biofilm resistance have been linked to efflux systems embedded in the membrane (22–25). Regulation of membrane proteins is a common resistance strategy. Analysis of the P. aeruginosa membrane proteome found a strong association between the abundance of certain outer membrane efflux transporters and the degree of resistance to ampicillin, kanamycin, and tetracycline (18). These results suggest that among the outer membrane protein markers found, some appear to be upregulated across antimicrobial classes while other markers show more regulation specificity, which is dependent on the specific antimicrobial substrate, hence making a case for the future investigation of shared homologous targets.

### Efflux pumps are a focus of resistance treatment due to their substrate promiscuity.

**FIG 1:**
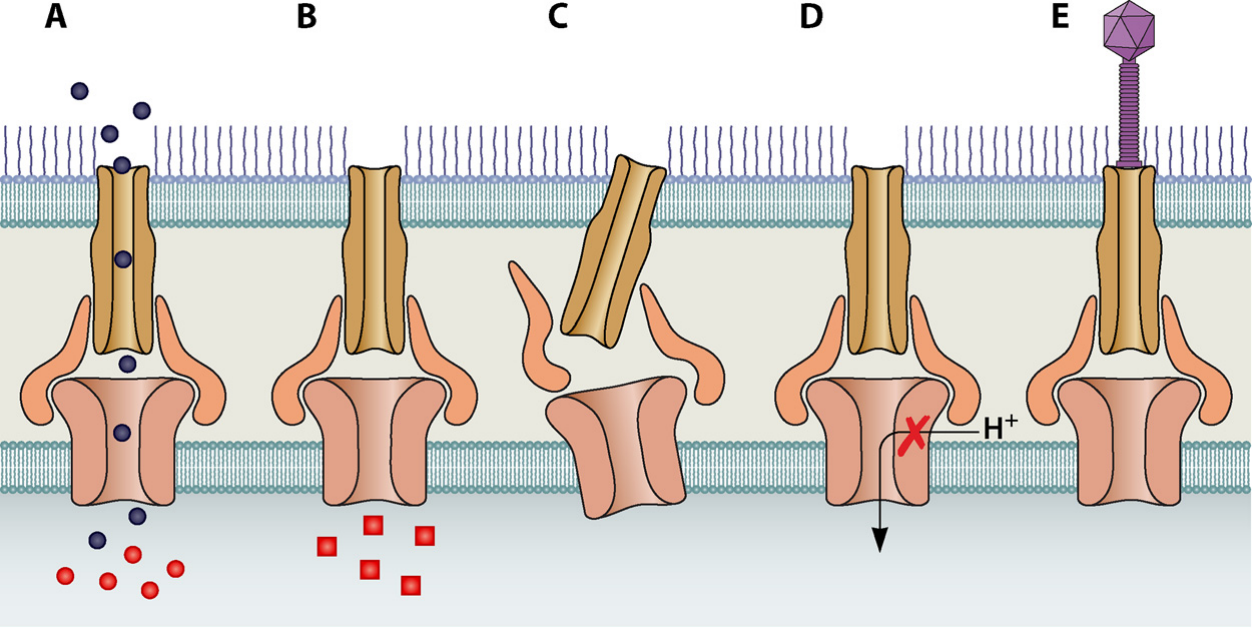
Efflux pump inhibitor (EPI) mechanisms (A to D) compared to phage steering (E). The figure illustrates (A) substrate competition causing efflux of EPIs (black circles) while retaining antimicrobials (red circles), (B) altered antimicrobial structure by EPIs (red squares) prevents recognition by the efflux system, (C) disruption of pump assembly, (D) disruption of the proton motive force required for efflux, and (E) phage steering using the outer efflux protein as a receptor to block efflux in addition to actively destroying pathogens.

Efflux systems located in the cell membrane have both generalized and specific responses to antimicrobials. P. aeruginosa possesses multiple efflux pump systems that have been extensively characterized, including MexAB-OprM, MexCD-OprJ, and MexXY-OprM, which often promote resistance to multiple antimicrobials (26–28). Both pharmaceutical and biological methods for efflux inhibition have been investigated. This includes efflux pump inhibitors (EPIs), which prevent removal of antimicrobials through methods such as downregulation of efflux pump expression, efflux competition, or blocking the outer membrane channel (Fig. 1A to D). Efflux competition between the EPI Phe-Arg-β-naphthylamide (PAβN) and antimicrobials leads to PAβN being preferentially pumped out and allows antimicrobials to reach lethal concentrations (29). Other EPIs such as spermine are thought to physically block the membrane channel of OprD in P. aeruginosa (30).

EPIs can have knock-on effects that further reduce pathogenicity. For example, efflux inhibition in P. aeruginosa and other pathogens affected quorum sensing-dependent virulence factors (31), invasiveness (32), and biofilm growth (25, 33), which could further increase antimicrobial efficacy. While EPIs are a potential method to combat resistance, some research outcomes highlight the question of whether efflux pumps are always a main driver of P. aeruginosa resistance (34, 35). Additionally, both EPIs and antimicrobials require continued development in response to the evolution of bacterial resistance.

### The evolutionary context underlying AMR is important to develop treatments.

AMR can be considered a form of evolutionary rescue (36). Evolutionary rescue occurs when adaptation allows a declining population to recover and avoid extinction (37). Treatments that minimize the probability of evolutionary rescue are expected to be the most effective (38).

The probability of rescue can increase due to existing mutations within a population, periods of environmental quality restoration, and gradual decay of environmental conditions (36). Additionally, extreme environmental changes, such as an application of concentrated antimicrobials, can be beneficial for the survival of mutants within the population (39). The role of migration, or horizontal gene transfer in antimicrobial resistance, can also favor evolutionary rescue, as seen by the transfer of a plasmid containing a β-lactamase gene from one Escherichia coli strain to a β-lactam-susceptible strain, allowing the latter to survive high levels of ampicillin (40).

Greater population size and initial genetic diversity have also been correlated with a higher likelihood of antimicrobial resistance evolution (41). Maintenance of genetic diversity through multiple adaptations moving as a “soft sweep” through a population may also increase the likelihood of evolutionary rescue (42). Diversity could be one predictor of drug efficacy, with diverse populations being more likely to survive (42).

Survival through evolution is a trait of not only bacteria but also their phage predators. Infection of “suboptimal” bacterial hosts by cyanophages led to diverse evolutionary outcomes in phage, which suggested that host availability affected bacteriophage evolution (43). The coevolution of phage and bacteria is a central concept behind the development of bacteriophage therapy.

## THE PRINCIPLE OF BACTERIOPHAGE THERAPY AND ITS PLACE IN MODERN MEDICINE

### Phage and bacteria coevolve, with host efflux pumps often being used for recognition.

Bacteriophages are DNA or RNA viruses that infect specific bacterial hosts through recognition of one or multiple receptor binding proteins (RBPs) on the cell surface, including efflux systems (44). Efflux pump proteins are the RBPs for multiple phages. Examples include P. aeruginosa phage OMKO1, which uses the M protein (45), Vibrio cholerae phage VP3, and E. coli phages U136B and TLS, which use the TolC protein as a coreceptor (46–48). TolC is also a receptor for the ST27, ST29, and ST35 bacteriophages, which are active against several Salmonella serovars, many with high TolC sequence similarity (49). However, despite TolC sequence similarity in some *Enterobacteriaceae*, the ST27, ST29, and ST35 are inactive against many *Enterobacteriaceae* species, demonstrating the host specificity of phages (49).

In response to phage predation, bacterial hosts have evolved both broad and specialized strategies to resist infection through inhibition of phage adsorption, injection, or replication. Adsorption can be prevented through biofilm production, as observed in biofilm protection of embedded Staphylococcus epidermidis from phage *Sepunavirus* phiIBB-SEP1 (50). Additionally, adsorption can be blocked by RBP modification, even while maintaining antimicrobial efflux dependent on the same protein (47). RBP mutations not only block phage attachment, but can also confer cross-resistance to multiple phage species targeting the same binding site (51). Furthermore, several phage resistance mechanisms can coexist, as observed in E. coli O157:H7 where exposure to phage PP01 changed in both the outer membrane lipopolysaccharides and outer membrane protein C channel expression (52).

While bacteria evolve to resist attack, phage coevolve to overcome defense mechanisms. Parallel evolution of four cloned bacteriophage PP01 populations converged to have the same point mutations, which improved binding to the E. coli O157:H7 outer membrane protein C receptor (53). Phages can even influence host quorum sensing to increase the ease of infection, as demonstrated by phage DMS3 lysogeny in P. aeruginosa that inhibited quorum sensing-controlled biofilm production, swarming behavior, and even anti-phage defenses (54, 55). The ability of phage to evolve in response to bacterial resistance is one reason bacteriophage treatment is appealing compared to fixed antimicrobial formulas.

### Resistance to bacteriophage and antimicrobials can be synergistic or antagonistic.

An appealing aspect of phage therapy is that it uses the host specificity that phages naturally possess to target pathogens of interest. However, in the context of divergent evolution of pathogens, this specificity is a double-edged sword. A substantial barrier to effective phage therapy is understanding how pathogens evolve resistance, both to phage and antimicrobials.

Biofilm formation is one resistance mechanism that could provide protection against both phage and antimicrobials. P. aeruginosa PAO1 was resistant to phage 14/1 in sublethal concentrations of gentamicin (56). The outcome was hypothesized to be a result of the generalized resistance promoted by biofilms (56), which commonly develop in sublethal antimicrobial conditions (57, 58).

Although biofilm formation can inhibit both antimicrobials and phage activity, some studies have identified certain phage enzymes as potential biofilm treatment agents. P. aeruginosa biofilms experienced a 99% reduction in bacterial exopolysaccharide viscosity through exposure to phages producing polysaccharide depolymerase (59). An enzyme produced by phage PT-6 hydrolyzed biofilms of P. aeruginosa strains isolated from patients with cystic fibrosis, a finding which could ultimately improve patient outcomes by disrupting biofilm growth (60). Additionally, biofilm degradation using P. aeruginosa phage LKA1 lysate did not affect ciprofloxacin or gentamicin activity (61), which is an important factor to consider when combining phage and antimicrobial treatment. Thus, phage enzymes directed toward biofilm degradation could become important auxiliary aids in bacteriophage therapeutics. However, due to the complexity of bacterial biofilm structures, phage enzymes require additional research to investigate their potential clinical applicability.

## THE DUAL ROLE OF EFFLUX PUMPS IN ANTIMICROBIAL RESISTANCE AND PHAGE SUSCEPTIBILITY

Bacteriophage steering deliberately uses the coevolution of phage and their hosts to treat bacterial infections (62). There have been multiple studies focused on phage steering of OMKO1 in P. aeruginosa (9, 45, 63). In one therapeutic investigation, phage OMKO1 directed evolutionary changes in multidrug-resistant P. aeruginosa to make it susceptible either to phage or to one of four antimicrobials of different classes (63). Through downregulation of the efflux system protein OprM, which OMKO1 uses as a receptor, the bacteria developed resistance to OMKO1, but also became susceptible to antimicrobials (Fig. 1E) (63). In computational modeling of *in vitro* phage-antimicrobial synergy, combination therapy of antimicrobials with phage OMKO1 against P. aeruginosa resistant to either phage or antimicrobials resulted in a treatment efficacy higher than that of either therapy individually (64). Successful therapeutic application of OMKO1 to treat a chronic P. aeruginosa infection in a patient demonstrated that prior knowledge of the evolutionary resistance pathways against antimicrobials and phage is critical for positive outcomes (45). Outcomes can be improved using screening methods, such as intentional overexpression of outer membrane proteins, to identify phage that target the ideal host receptor to introduce antimicrobial susceptibility (49).

Other phage steering mechanisms have been identified, such as the use of Acinetobacter baumannii phages ΦFG02 and ΦCO01 (65). Phage resistance resulted in bacterial capsule loss and subsequent susceptibility to certain antimicrobials and other phages (65). An earlier study found that Acinetobacter baumannii with multidrug resistance to more than three antimicrobials, either within or across classes, were significantly more likely to be phage susceptible, with phage infection rates over 80% compared to around 50% for cells with no AMR (66). However, due to the multitude of resistance strategies, including enzyme production, and the possibility that some anti-phage adaptations could still maintain AMR (46), application of a single phage in conjunction with a single antimicrobial is unlikely to be the most effective treatment. Treatment combining ϕPA01 and ϕPA02 in a phage cocktail suppressed P. aeruginosa growth for 20 h compared to between 8 and 9 h for either phage applied individually (67). Furthermore, the use of the phage cocktail combined with either ciprofloxacin or meropenem was even more effective than the cocktail alone and suppressed growth for 96 h (67). Designing phage cocktails to contain multiple phages that steer evolution toward antimicrobial susceptibility would improve phage therapy outcomes. The evolutionary design is in contrast to selection of phages based only on host specificity or growth inhibition, which are some of the most common methods currently used to choose cocktail candidates (68, 69).

### Bacteriophage as a therapeutic agent faces production and delivery challenges.

Current limitations to bacteriophage therapy include the lack of purity and stability in solution as well as the reduction in concentration between the point of administration and the site of bacterial infection (70). Phage purification has been achieved using naturally occurring phage aggregation and microfiltration (71, 72). Microencapsulation of precipitated phages has been suggested to protect phages from stomach acid after oral administration (73). As the number and availability of phage products increase, production standards and regulatory frameworks must be created to provide the same safe outcomes and ease of access expected of antimicrobial therapeutics (74–76).

Successful clinical outcomes using phage treatment have been reported (45, 75, 77, 78), but to date there has been a lack of robust, double-blind phase III trials (76, 78). A double-blind phase I/II clinical trial for phage treatment of P. aeruginosa ear infections demonstrated reduced infection levels (79). Conversely, another double-blind phase I/II clinical trial that treated burn wounds using a P. aeruginosa phage cocktail cream resulted in slower recovery compared to that of conventional treatment (80). Ideal treatment schedules also remain relatively unexplored, although one study found that the application of streptomycin 12 h after phage led to the greatest reduction in P. aeruginosa POA1 density *in vitro* (81). Mixed outcomes in studies are caused by wide variations in experimental design, including phage species used, concentrations of phage or antimicrobial applied, application methods, and timing of therapy. To adopt phage therapy, it is imperative that more high-quality clinical trials are undertaken and barriers to implementation are resolved.

## DISCUSSION

Evolution continues to play a central role in the rise of antimicrobial resistance. If resistance is a form of evolutionary rescue, phage steering can decrease the probability of rescue. Rather than simply identifying phages that target bacteria of interest, *in silico* experiments and screening to invoke evolutionary pathways as well as direct treatment to specifically inhibit the evolution of resistance will bring a more directed approach to phage therapy (82, 83). Phage steering with antimicrobials has been investigated in only a few host-phage combinations (46, 63, 65). Expanding the host strains and types of phage included in studies will lead to a greater understanding of phage steering applications as well as phage-antimicrobial synergies (84, 85). Phages are advantageous due to their abundance, natural antibacterial activity, bacterial specificity, and ability to evolve. Ultimately, a better understanding of phage ecology and evolution will establish phage steering as a major tool for clinicians and researchers to not only combat AMR, but further explore the mechanisms of resistance development.
